# Empirical Tryout of a New Statistic for Detecting Temporally Inconsistent Responders

**DOI:** 10.3389/fpsyg.2018.00518

**Published:** 2018-04-10

**Authors:** Matthew J. Kerry

**Affiliations:** Department of Management, Technology and Economics, The Swiss Federal Institute of Technology, Zurich, Switzerland

**Keywords:** repeated measures, test–retest, insufficient effort responding, survey research, longitudinal, time

## Abstract

Statistical screening of self-report data is often advised to support the quality of analyzed responses – For example, reduction of insufficient effort responding (IER). One recently introduced index based on Mahalanobis’s D for detecting outliers in cross-sectional designs replaces centered scores with difference scores between repeated-measure items: Termed person temporal consistency (*D*^2^_ptc_). Although the adapted *D*^2^_ptc_ index demonstrated usefulness in simulation datasets, it has not been applied to empirical data. The current study addresses *D*^2^_ptc_’s low uptake by critically appraising its performance across three empirical applications. Independent samples were selected to represent a range of scenarios commonly encountered by organizational researchers. First, in Sample 1, a repeat-measure of future time perspective (FTP) inexperienced working adults (age >40-years; *n* = 620) indicated that temporal inconsistency was significantly related to respondent age and item reverse-scoring. Second, in repeat-measure of team efficacy aggregations, *D*^2^_ptc_ successfully detected team-level inconsistency across repeat-performance cycles. Thirdly, the usefulness of the *D*^2^_ptc_ was examined in an experimental study dataset of subjective life expectancy indicated significantly more stable responding in experimental conditions compared to controls. The empirical findings support *D*^2^_ptc_’s flexible and useful application to distinct study designs. Discussion centers on current limitations and further extensions that may be of value to psychologists screening self-report data for strengthening response quality and meaningfulness of inferences from repeated-measures self-reports. Taken together, the findings support the usefulness of the newly devised statistic for detecting IER and other extreme response patterns.

## Introduction

There are many reasons to expect score fluctuations in repeated-measures designs (Thorndike, 1951). Incumbent on all organizational researchers is identification of biasing responses to self-report measures, defined by Paulhus (2002) as, “Any systematic tendency to answer questionnaire items on some basis that interferes with accurate self-reports” (p. 49). One particular form of biased responding that has received recent research focus is careless or insufficient effort responding (IER), which refers to general inattentive or effortless responses (Huang et al., 2012). Due to IER’s quality reduction and high prevalence (∼15%; Meade and Craig, 2012), various strategies are currently used to help researchers address IER. In a first example, direct-design strategies (e.g., validation/bogus items) involve *a priori* assessment-design features for deliberately gauging response quality via direct-reporting by respondents. In a second example, archival para-measure strategies (e.g., response times) involve unobtrusive, *post hoc* inspection of metrics generated by respondents’ administrative-proceeding through assessments. In addition to direct design and archival measure options, a popular, third option of statistical-analytic strategies (e.g., Mahalanobis *D*) involves researcher computational calculation of indicators for IER detection or severity (indexing). The statistical-analytic approach has generated several tools for screening data to index IER (e.g., Meade and Craig, 2012).

This third strategy of statistical-analytics is the focus of the current study. Specifically, the currently study seeks to evaluate the performance of a newly developed analytic-tool (referred throughout as “D_ptc_”) by screening data for potential IER using three application samples. Before examining D_ptc_’s empirical performance, first, its conceptual contribution is presented below by delineating D_ptc_’s relation in a framework of extant statistical-analytics for detecting IER.

### Extant Framework of IER Statistical-Analytics

Extant IER statistical-indices have largely been limited by formulations rooted within classical internal reliability principles. For example, the Mahalanobis’s (1936) indicates outlier severity in a cross-sectional sample, i.e., between-person. Whereas Jackson’s (1976) personal reliability coefficient is premised on within-person consistency, it is computed using internal split-half reliabilities (within-assessment). A more recent example of within-person consistency, which proposes simply computing the standard deviation of responses over latest-appearing items, suggests that the methodological landscape remains rooted in classical conceptions and restricted to cross-sectional designs (Dunn et al., 2018).

Perhaps unsurprisingly, the methodological rigidity of IER indices limited by internal-reliability principles is evidenced in more substantive researches (Bowling et al., 2016). For example, in a repeat-measures study of IER as a substantive person-confound, personality researchers found that distinct IER indices showed stronger convergent correlations within-measurement occasion (*r* = 0.62) than identical-IER indices’ between-measurement occasions, i.e., over time (*r* = 0.42), *z*_(1)_ *=* 2.50, *p* = 0.012 (Bowling et al., 2016). This finding may be interpreted as more inconsistency across measurement occasions than within a single-measurement occasion.

**FIGURE 1:**
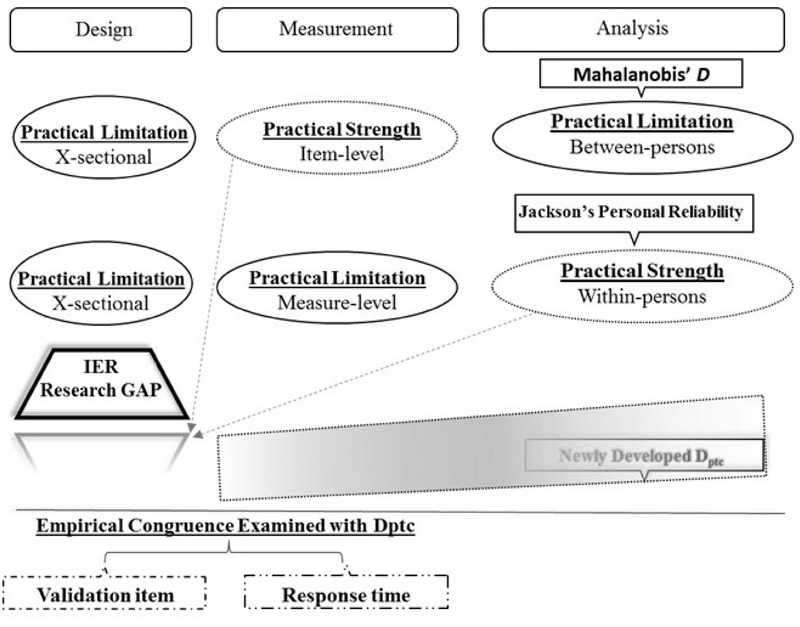
Relational-framework illustrating IER research gap filled by new D_ptc_ tool.

Taken together, a need for an IER index unbound by cross-sectional designs and appropriate for repeat-measure applications is reasonably identified. Furthermore, **Figure 1** below illustrates the current gap in extant IER analytical tools. In the next section, below, D_ptc_’s recent development to fill the gap in IER research is reviewed followed by the current study’s contribution with empirical evaluation of D_ptc_ across three sample applications.

### Recent IER Development and Current Contribution

Just over 3 years ago, DeSimone (2014) introduced a novel statistic (*D*^2^_ptc_) designed to detect IER specifically in repeated-measures data. Conceptually, the *D*^2^_ptc_ integrates principles from two former indices of reliable response patterns, (1) Jackson’s (1976) personal reliability coefficient and, perhaps more familiar, (2) Mahalanobis’s (1936) distance score (Mahalanobis D). A full account of its computation is beyond the scope of this study, but its’ central premise rests on substituting ‘centered’ values in the original Mahalanobis D formula with ‘individual-difference scores’ between two assessment occasions. For further information, readers are referred to DeSimone (2014), but its formula is given below,

$$D_{\mathrm{ptc}}\;=\;\;\sqrt{{\left(\vec{X_{i(\mathrm{t1})}}\;-\vec{\;X_{i\left(\mathrm{t2}\right)}}\right)}_{\;}^{T}DIF_{\mathrm{xx}}^{-1}\;(\vec{X_{i\left(\mathrm{t1}\right)}}\;-\;\vec{X_{i\left(\mathrm{t2}\right)}})}$$

where 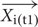 and 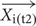 indicate the response vectors for participant *i* at times (t1) and (t2), respectively. *DIF*^-1^_xx_ is defined as the inverted covariance matrix with difference-scores. As a multivariate distance indicator based on raw-response patterns over time (*and* within-individual), D_ptc_ could become a powerful tool for strengthening the quality of within-person datasets. For example, considering Curran’s (2016) recommended multi-hurdle sequential methods approach to IER, D_ptc_ can be adapted for use with the first two methods recommended for IER screening (e.g., response time, longstring analysis). As with the original Mahalanobis D, *D*^2^_ptc_ is asymptotically distributed as a chi-square statistic (*X*^2^). This permits statistical tests of significance with the degrees of freedom (*df*) set equal to the number of scale items. For example, the *D*^2^_ptc′s_ introductory simulation (detailed further below) generated hypothetical responses to a 5-item scale (*j* = 5), and *D*^2^_ptc_ scores were significant (respondents flagged for inconsistency) if they exceeding the 95% significance level (*p* < 0.05) of *X*^2^ with *df* = 5; That is, responders were flagged as potentially inconsistent if their *D*^2^_ptc_ scores > *X*^2^(5) = 11.07.

Initial performance of the *D*^2^_ptc_ was evaluated on simulation data and supported its viability for detecting IER (DeSimone, 2014). Specifically, using a simulated dataset of *N* = 30 respondents with *n* = 5 random respondents and *n* = 5 extreme-inconsistent respondents, the *D*^2^_ptc_ index successfully detected (flagged) *n* = 3/5 of random respondents and all (*n* = 5/5) of the extreme respondents. Despite its viability in simulated data, subsequent uptake appears to be absent. This is surprising given the increased research attention to data response-quality, as well as the prevalence of large-scale panel data with repeated-measures designs.

Having overviewed the conceptual framework, computational formulae, and simulation performance of the *D*^2^_ptc_ index, the present study further examines *D*^2^_ptc_’s empirical performance across three, independent samples. For each sample application, the relevant construct is introduced, hypotheses are formulated, sample characteristics are summarized, and test results of *D*^2^_ptc_’s first-empirical performance evaluations for detecting temporal inconsistent response patterns are presented.

### Ethical Considerations for Empirical Evaluations

All analyses in this paper constitute secondary analyses of publicly available or de-identified data, which does not meet regulatory definitions of “research with human subjects,” ergo, is exempted from IRB review. The first data is publicly available, and the second and third datasets are non-identifiable. The non-identifiable datasets (two and three), however, received IRB approvals for original, primary study from review committees at Emory University and the Georgia Institute of Technology, respectively. That is, participants were given informed consent in the original, primary dataset collections for the current study’s secondary analyses.

## Application 1 – Individual Temporal Inconsistency

In light of the aging workforce, a likely relevant and useful demonstration of the *D*^2^_ptc_ may begin with its application to a sample of experienced workers and retirees (age > 40-years). One construct that figures prominently in both organization and career research domains is FTP (Carstensen and Lang, 1996, Unpublished). A two-wave administration of the FTP instrument in a mixed sample of workers and retirees is the context for the first empirical application of *D*^2^_ptc_.

Many postulated causes of IER is ostensibly relevant to aging (see Knäuper et al., 2016). For example, Meade and Craig (2012) identified response fatigue and decreased social contact as two causes of IER. The prevalence of inconsistent responding as a function of age has also received empirical support (Colsher and Wallace, 1989). The author of the *D*^2^_ptc_ index postulated that it is likely to, “fare better in the identification of inconsistent responders than random responders” (DeSimone, 2014; p. 18). Taken together, thus, the first hypothesis for *D*^2^_ptc_’s first-empirical application is stated as,

***H1:*** *D*^2^_ptc_ will be positively associated with chronological age.

The statistical-equivalence of procedures for assessing measurement invariance across groups and over time is fairly well-known (Meredith, 1993). As substantive interest in IER has recently increased, then, so has interest in IER’s temporal endurance, i.e., behavioral consistency of persons across situations and over time (Funder and Colvin, 1991). The temporal stability of IER has recently received empirical support (Bowling et al., 2016).

Extrapolating, it is arguable that cross-sectional indicators of inconsistent responding, such as reverse-scored (RS) items should correspond with *D*^2^_ptc_ as an analogous index designed for applications in repeated-measure designs (see Schmitt and Stults, 1986). Recent empirical evidence supported IER’s temporal stability over a 13-month repeated-measures design, which further corresponded to baseline indices (Bowling et al., 2016). Because the FTP instrument comprises both RS and standard-scored (SS) items, this postulate is directly tested in the current data; That is, SS items are keyed to be scored in the direction presented, with higher response categories scored as higher levels on the focal trait, whereas RR items are RS, with higher response categories corresponding to lower levels on the focal trait. Restated, the second hypothesis for *D*^2^_ptc_ is:

***H2:*** *D*^2^_ptc_ will detect more temporally inconsistent respondents to RS_(j__=__3)_ than SS_(j__=__7)_ items.

### A1 Data

Matched two-wave data on the FTP instrument was obtained from RAND’s American Life Panel (ALP). Original collection occurred from December 2011 – August 2014. A subsample (*N* = 620) received a follow-up questionnaire approximately *M* = 26.65 days later. FTP administration constituted a common-item non-equivalent groups design. The non-equivalent groups admits the possibility of other sample composition or person confound covariates as potential mechanisms for further examining of any substantive findings regarding temporal inconsistency reported in the current, empirical-methodological demonstration study.

### A1 Results

Applying the *D*_ptc_ formula to the total *j* = 10-item FTP measure^1^ resulted in *N* = 94 of 620 participants (∼15%) being flagged for temporal-inconsistent responding, *X*^2^_(10)_ = 18.31, *p* < 0.05. For practical implications, it may be noteworthy that *D*^2^_ptc_ values were significantly associated with job status (employee < retiree), *X*^2^_(1)_ = 8.45, *p* < 0.001.

Regarding Hypothesis 1 for greater temporally inconsistent responding with age, the *D*^2^_ptc_ values, as indicators of temporal inconsistency, was significantly associated with chronological age, *r* = 0.19, *p* < 0.001. The mean-age difference between stable (*M* = 59.74) and unstable (*M* = 63.49) responders was also statistically significant, *t*_(618)_ = -3.40, *p* < 0.01. These findings support Hypothesis 1.

Regarding Hypothesis 2 for greater temporally inconsistent responding to RS compared to SS items, *N* = 55 respondents were flagged with a significant D_ptc_ value, *X*^2^_(3)_ = 7.81, *p* < 0.05. By comparison, *N* = 9 respondents were flagged with a significant D_ptc_ value based on SS items, *X*^2^_(7)_ = 14.07, *p* < 0.05. The proportional equivalence based on a ‘2×2 contingency’ Pearson chi-squared test was rejected, *X*^2^ = 34.86, *p* < 0.001. This finding supports Hypothesis 2. After removing *D*^2^_ptc_-flagged participants, as expected, the average-item temporal consistencies increased for both SS (*r* = 0.49 → 0.59) and RS items (*r* = 0.37 → 0.44).^2^

## Application 2 – Team Temporal Inconsistency

The second application of the *D*^2^_ptc_ index is an extension from individual-level repeated measures to team-level repeated measures. An extensively researched construct, team efficacy (TE), is the focus for *D*^2^_ptc_’s second application.

Bandura (1997) defined TE as “a group’s shared belief in its conjoint capabilities to organize and execute the courses of action required to produce given levels of attainments” (p. 477). Mirroring process- and outcome-efficacy distinctions at the individual-level, TE has been similarly distinguished and empirically supported (Collins and Parker, 2010). Specifically, team-process efficacy (TPE) has been defined as a team’s belief that they can successfully function with its members, such as interpersonal cooperation, communication, and collaboration. Team-outcome efficacy (TOE) refers to a team’s confidence level that they can achieve a specific level of performance.

Based on DeRue et al.’s (2010) taxonomy of efficacy dispersion, in teams where membership remains constant and there is a high level of interdependence, both TPE and TOE should show convergence (within-team agreement) over time as experience increases. In the current sample, however, intact teams were rotated to new clinical performance tasks with limited feedback. We might expect, then, that task-related TOE should be less stable than person-related TPE. Thus, it is hypothesized:

***H3:*** *D*^2^_ptc_ will be more strongly associated with TOE compared to TPE.

Group development scholars have posited that the condition of a team’s formation has enduring impact on team processes (McGrath, 1984). In the efficacy domain, theorizing by Gibson and Earley (2007) predict that initial aggregate beliefs serve as a context, with higher levels corresponding to a stronger context that affords members more accurate perceptions of team capabilities. This reasoning parallels personality scholar’s recent, substantive interest in IER. That is, IER is argued to be jointly determined by stable-dispositional person attributes and contextual cues (i.e., behavioral consistency *vis-à-vis* contextual strength). In the current study, we examine if higher team-agreement on TPE at baseline may operate in accordance to a stronger ‘context’ for members reporting their beliefs. Following this rationale, it is tenable that higher baseline agreement is associated with higher temporal consistency. Specifically, it is hypothesized:

***H4:*** *D*^2^_ptc_ will be higher for teams with low baseline-agreement_(k__=__12)_ on TPE than teams with high baseline-agreement_(k__=__12)_.

### A2 Data

*N* = 183 medical and nursing students, participating in an interprofessional education (IPE) session, were randomly allocated to *k* = 24 mixed-profession teams. Following a 1-h didactic, teams completed three consecutive high-fidelity clinical simulations. One faculty member was assigned to facilitate each team (fixed to particular clinical simulation). Faculty introduced and instructed teams on each case simulation, and teams received structured feedback from faculty on teamwork competencies at the end of each case simulation. Teams then rotated to a new case simulation with a new faculty facilitator. Pre-simulation, after faculty introduced teams to the clinical case, students completed *j* = 8 items assessing TOE and TPE using a referent-shift model. Items were adopted from Collins and Parker (2010). Repeat-administration occurred post-feedback for teams’ current clinical simulation. A total of six completed forms were collected for each student (*M* team-size = 7 members). To maximize expected agreement over time, TE scores from the last two trials are examined (assessments five and six).

### A2 Results

Regarding Hypothesis 3 for more temporally stable responding associated with TPE compared to TOE, the TPE – *D*^2^_ptc_ correlation (*r* = 0.48) was significantly greater than the TOE – *D*^2^_ptc_ correlation (*r* = 0.15), *z*_(1)_ *=* 1.83, *p* = 0.03. Additionally, *k* = 2/24 teams were flagged as temporally inconsistent (∼8.33 %) based on TOE scores, whereas *k = 0*/24 teams were flagged (0%) based on TPE scores taken together, these findings support Hypothesis 3.

Regarding Hypothesis 4 for more temporal inconsistency for low-agreement TPE teams compared to high-agreement teams, an independent sample *t*-tests was conducted on TPE median-splits of baseline R_wg_ scores. The mean-*D*^2^_ptc_ difference between high-agreement (*M* = 3.03, *SD* = 1.92) and low-agreement (*M* = 4.64, *SD* = 2.45) teams was statistically significant in the hypothesized direction, *t*_(22)_ = 1.49, *p* = 0.04. These findings support Hypothesis 4.

## Application 3 – Experimental Prevention of Temporal Inconsistency

Some of the earliest IER research was conducted in the context of personality assessments (Greene, 1978). Despite IER’s persistent challenge to substantive personality research, DeSimone notes that D_ptc_’s methodological flexibility enables versatile use across distinct study designs. Beyond D_ptc_’s original purpose for detecting repeated-measures inconsistent responding, DeSimone (2014) posited extension of D_ptc_ with exemplification in intervention studies, “*D*^2^_ptc_ could be used to identify the participants whose scores changed the most (or changed to a statistically significant degree)” (p. 8).

Building on D_ptc_’s postulated, design-flexible multifunction potential, the third and final Application evaluates an extension of D_ptc_ from repeated-measure to a randomized-control experiment design. Specifically, reasoning from experimental random-assignment, D_ptc_ is evaluated as a potential index for experiments that may seek to comparatively evaluate the effectiveness of different IER-prevention or reduction strategies. For example, empirical evidence has indicated that sensitive survey content is significantly associated with IER rates (Camus, 2015, Unpublished). The logic of content-responsitivity (participant engagement via more stimulating or relevant focus measures) is consistent with earlier-described findings regarding survey fatigue/response burden from tedious and unvaried questionnaire content, such as systematic directional scoring of items (e.g., uniform use of SS items).

In the current experiment, subjective-life expectancy (SLE) questions presented before Big-Five personality factors are argued to decrease Big-Five IER by raising respondent interest through more engaging SLE items. A tenable mechanisms for expecting such effects come from empirical findings that have identified mortality salience as a determinant of motivated responding, such as trust beliefs (Paulhus, 1983). In turn, it is expected that the cognitive context of SLE-items will systematically prime more effortful responses via increased content responsitivity (Nichols et al., 1989). Increased content responsitivity should be expected to reduce temporally inconsistent responding. Hypothesis 5 is stated as,

***H5:*** SLE-treatment groups will exhibit less inconsistency (*D*^2^_ptc_) compared to Controls.

The last hypothesis formulated to test *D*^2^_ptc_ pertains to its congruency with other established IER indices. This accords with Messick’s (1995) advisory for examining structural validity, such that scores should be “rationally consistent with what is known about the structural relations inherent in behavioral manifestations of the construct in question” (p. 746). From DeSimone et al. (2015) taxonomy of data screening techniques, *D*^2^_ptc_ appropriated as an analytical-statistic strategy. Therefore, the final hypothesis pertains to *D*^2^_ptc_’s congruence with two tools (validation item/response time) representative of the other remaining complementary strategies (direct-design/archival para-measure) from the Section “Introduction” and illustrated in **Figure 1**. Specifically, hypothesis 6 is stated as:

***H6:*** *D*^2^_ptc_ will exhibited convergent-correlates with instructed items (direct-design) and response time (archival para-measure) as corroborative indices of quality response data.

### A3 Data

A simple random sample was collected from respondents to the online recruiting platform, Mechanical Turks (*N* = 109). Participation was incentivized with monetary compensation ($.50) for successful completion of the online questionnaire. After reporting demographics, participants were randomly assigned to the three experimental conditions. *N* = 39 participants were allocated to the control arm, *N* = 34 participants were allocated to the ‘Live-to’ treatment arm, and *N* = 36 participants were allocated to the ‘Die-by’ treatment arm. The average-completion time for the questionnaire was estimated at *M* = 13.98 min.

### A3 Results

Preliminary evidence supported the effectiveness of the experimental manipulation, such that participants in the ‘Live to’ condition reported a 10.02% greater likelihood of living to age 80-years compared to participants in the ‘Die by’ condition, *M* = 1.02 (0.22), *t*(108) = 4.581, *p* < 0.001.

**FIGURE 2:**
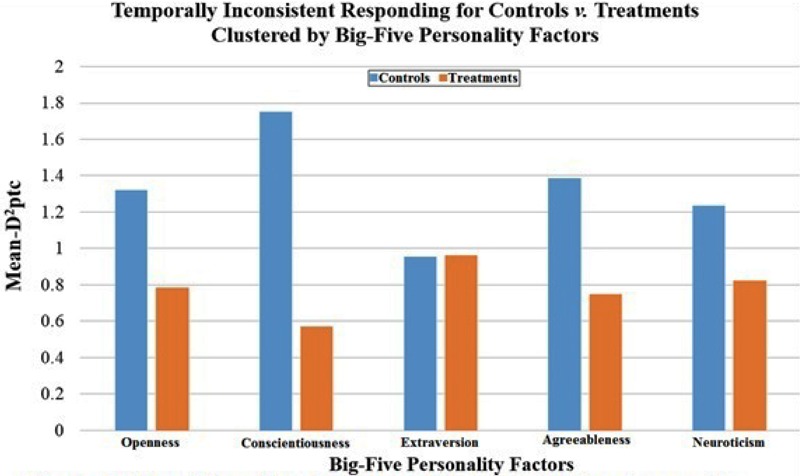
Mean-*D*^2^_Ptc_ values indexing experimental effects on temporally inconsistent responding across the five-factor model of personality, *N* = 109.

Regarding Hypothesis 5 for the treatment’s reduction of inconsistent responding, *D*^2^_ptc_ values were in the hypothesized direction across all five factors, but only reached statistical significance for three factors. Specifically, the *D*^2^_ptc_ index was significantly lower for Treatments than Controls on personality factors Openness (*M*_Dif_ = 0.54 [0.34], *t*_(101)_ = 1.53, *p* = 0.065), Conscientiousness (*M*_Dif_ = 1.12 [0.42], *t*_(88)_ = 2.70, *p* = 0.008), and Agreeableness (*M*_Dif_ = 0.72 [0.41], *t*_(88)_ = 1.77, *p* = 0.065). Clustered bar-charts for all five factors are displayed in **Figure 2** below.

Aggregating across the five personality factors, *N* = 17/155 (11%) of participants in the Control condition were flagged as temporally inconsistent responders, compared to *N* = 13/310 (4%) in the treatment conditions. The proportion was significantly different (Pearson’s *X*^2^_(1)_ = 7.85, *p* < 0.05) and supports the reduction of temporally inconsistent responding for the aggregated treatment, compared to controls. Notably, the proportionally lower inconsistent responding in treated participants converged with a significantly higher completion rate (96%) compared to control participants (30%), Pearson’s *X*^2^_(1)_ = 9.03, *p* < 0.01. Taken together, these finding provide partial support for Hypothesis 5.

Regarding Hypothesis 6 for convergent-correlates between *D*^2^_ptc_ and complementary tools representative of two other IER strategies, findings supported congruence of *D*^2^_ptc_ with, both the direct-design strategic tool (validation items), as well as the archival para-measure strategic tool (response times). Specifically, validation item success rates were higher with more temporal consistency, *r*_(*D*^2^ptc-response success)_ = -0.29, *p* < 0.04. Regarding response times, surveys took longer to complete with more temporal consistency, *r*_(*D*^2^ptc-duration)_ = -0.21, *p* < 0.05.

These findings conform to previous reports of established IER indices and support the nomological concordance of *D*^2^_ptc_, thus, Hypothesis 6 is supported.

## Discussion

Recently, personality researchers have reframed their interest in IER from that of mere “methodological nuisance” to one of a substantive variable of interest (Bowling et al., 2016; p. 218). Similarly, in the current study, a novel statistical tool (*D*^2^_ptc_) for detecting IER in repeat-measures designs was critically appraised for substantive use with empirical performance evaluation across three independent sample applications. Test results of the hypotheses evaluating *D*^2^_ptc_’s first-empirical applications are summarized below.

In the first application, support was found for *D*^2^_ptc_’s positive associations with chronological age (H1), which corroborates past reports of inconsistent responding in older adults. Support was also found for *D*^2^_ptc_’s identification of more temporally inconsistent responders based on RR items compared to SS items (H2).

In the second application, *D*^2^_ptc_ was observed to be sensitive to changes based on aggregated reports as indicators of team-level constructs. Specifically, in accordance with predictions from the TE literature, *D*^2^_ptc_ was more strongly associated with TOE compared to TPE in a sample of intact teams rotating through novel performance tasks (H3). Furthermore, supporting for the dynamic measurement of emergence in teams, *D*^2^_ptc_ was found to be related to baseline within-team agreement scores, such that teams with relatively more dispersion on TPE exhibited greater instability on mean-TPE over time (H4).

In the final application, it was hypothesized that the attribute framing manipulation of subjective life expectancies (SLE) would lead to differences in temporally inconsistent responding. H5 was generally supported, with the largest discrepancies in response consistency observed for personality factors Openness, Conscientiousness, and Agreeableness. Notably, this pattern of factor-specific relations is consistent with previous studies examining the substantive relations between personality and IER tendencies (Bowling et al., 2016). H6 supported *D*^2^_ptc_ congruence as a statistical index with established tools representing dissimilar strategies of direct-design (validation items) and archival para-measures (response times). Despite generally supportive findings from *D*^2^_ptc_’s first-empirical applications, some important limitations to the current study are noted below with complementary considerations for future research directions for extending *D*^2^_ptc_ development.

### Practical Implications

Toward building on *D*^2^_ptc_’s empirical support with practical implications for future extensions, it warrants noting that the computational formula of *D*^2^_ptc_ may denote a more accurate terming as an index of temporal agreement, rather than consistency. That is, *D*^2^_ptc_ is sensitive to absolute-value changes, rather than relative-rank ordering changes over time. This agrees with the *D*^2^_ptc_ developer’s recommended use as suitable for detection of temporal IER in the assessment of stable-traits rather than assessment of transient-states where temporal change is more reasonably expected. Extrapolating to subsequent studies by *D*^2^_ptc_’s developer that has identified the differential impact of two IER subtypes (random responding versus extreme-inconsistent responding), the *D*^2^_ptc_ should be expected to perform better in the detection of extreme-inconsistent response patterns. This follows general consensus among IER research for multi-strategic approaches by researchers for optimally addressing specific IER forms that may be appropriately detected/reasonably expected according to study design and focal research questions (Meade and Craig, 2012). One potentially powerful combinatory extension for *D*^2^_ptc_ may be realized by its methodological flexibility for multi-hurdle sequential IER strategies, such as that presented by Curran (2016). One illustrative example pertains to accommodating D_ptc_’s examinee-indicator’s classical-measurement scoring limitations with IRT-specified substantive response styles of interest. Similar to several IRT-scoring procedures forwarded on to more conventional classical analyses, IRT-specification of substantive response styles of interest or concern may generate estimates suitable for forwarding onward for subjection to D_ptc_’s classical testing appropriated for repeated-measure. In Appendix B, limited-IRT scoring after D_ptc_-flagged removal of ∼15% sample 1 suggested practically meaningful increase in stability (∼14.3%) with negligible power-reduction (<0.1%). This practically meaningful usage of D_ptc_-flagged removal/sample reduction is further supported in terms of specificity by classical-measurement indices of internal reliabilities (see, Appendix A for impact of D_ptc_-flagged removal on estimates based on classical measurement frameworks).

### Current Limitations and Future Directions

There are a few substantive limitations to the current empirical evaluation of D_ptc_’s performance that warrant note. First, as stated in D_ptc_’s original simulation study, its use relies on non-missing data (complete cases), and this likely limited current empirical evaluation in terms of sampling generalizability and analytical robustness under conditions of imputed-missing values. Second, despite D_ptc_’s item-level basis, more precise specifications for detecting IER are available through modern measurement theory approaches, such as item response theory (IRT) (Jin et al., 2018). Interested readers are also directed to Appendices B, C for a limited-IRT extension after removing D_ptc_-detected responders from sample 1’s first empirical application.

## Conclusion

The applications presented here were selected for variety of ostensibly familiar research scenarios encountered in the psychological sciences. They are non-exhaustive, and many provocative extensions of temporal inconsistency remains to be explored. For example, the *D*^2^_ptc_ is a normative indicator computed from differences scores, thus, researchers might consider how external cohort or period phenomena may systematically impact pre–post observations. The *D*^2^_ptc_ author suggested that, “There is no reason why techniques for assessing temporal consistency should not inform research assessing measurement invariance (and vice versa)” (p. 18). A common prerequisite for measurement invariance established between groups or over time is the setting of a scale. Extrapolating, future studies might consider *D*^2^_ptc_’s sensitivity to different time scales (or for setting an origin in repeat-measures design). On whole, given the performance of the *D*^2^_ptc_ in its first-empirical applications, the current author endorses its use as a unique tool developed specifically for identifying IER in repeat-measures data. Furthermore, *D*^2^_ptc_’s flexible application across observational and experimental designs supports *D*^2^_ptc_’s multifunction usage through versatile applications.

## Author Contributions

The sole-author of this work secured all secondary data for demonstration, conducted all analyses, and completed all technical and expository write-up.

## Conflict of Interest Statement

The author declares that the research was conducted in the absence of any commercial or financial relationships that could be construed as a potential conflict of interest.

## Footnotes

1 Please see Appendix A for an overview of significant *D*-value frequency/% of sample across all three applications and measures.

2 Please see online Supplementary Material for more information.
