# Supplementary material for: Empirical Tryout of a New Statistic for Detecting Temporally Inconsistent Responders
Source: Front Psychol. 2018 Apr 10;9:518. doi: 10.3389/fpsyg.2018.00518 (PMC5902740; doi:10.3389/fpsyg.2018.00518)
Supplement: Supplementary file 1 [file Data_Sheet_1.PDF]

Online Supplementary Material – APPENDIX A
Applications 1-3: Summary Sample Characteristics of D-value Prevalence

Table A - Samples A1-A3 | Summary D2ptc Prevalence by Application Sample Frequency (n) and Rates (%)

	Sample 1	Sample 2		Sample 3	
		Outcome-Eff	*Process-Eff*	*Treatments*	*Controls*
Total size (*N*)	*N*=620	*N*=24	*N*=24	*N*=310	*N*=155
Inconsistent (*n*)	*n*=96	*n*=2	*n*=0	*n*=13	*n*=17
Inconsistent rate (*%*)	*n*=15%	*n*=0%	*n*=5%	*n*=4%	*n*=11%

Note. Outcome-Eff = Outcome-Efficacy Measure; Process-Eff = Process-Efficacy Measure. Treatments = Sum of live-to (*n*=34) and die-by (*n*=36) treatment conditions.

Sample 1's 10-item FTP measure = $X^2_{(10)} > 18.31$ D^2_{ptc} critical value (c.v.)*;*

Sample 2's 4-item team-outcome and team-process efficacy measures = $X^2_{(4)} >$ D^2_{ptc} c.v.;

Sample 3's 5-item aggregated Big-Five single-item Factor measures = $X^2_{(5)} >$ D^2_{ptc} c.v.;

All D^2_{ptc} c.v.'s were assessed at conventional α = 95% significance levels ($p < .05$).
