# Supplementary material for: Empirical Tryout of a New Statistic for Detecting Temporally Inconsistent Responders
Source: Front Psychol. 2018 Apr 10;9:518. doi: 10.3389/fpsyg.2018.00518 (PMC5902740; doi:10.3389/fpsyg.2018.00518)
Supplement: Supplementary file 2 [file Data_Sheet_2.PDF]

Online Supplementary Material – APPENDIX B
Application 1 – Supplementary Exploratory Analyses

Bereiter (1963) articulated that as correlations between measures increase, the reliability of their difference scores decrease. Conversely, removal of 'temporally inconsistent' responders may also remove substantive change or, otherwise, reduce the internal reliability of measures. For example, inspection of the average test-retest correlations was significantly higher for SS items ($r = .49$) compared to RS items ($r = .37$), $z(1) = 2.53$, $p < .01$.

To inspect the possible decrement to internal reliabilities as a result of removing 'temporally inconsistent' responders, Cronbach alphas and test-retest correlations were computed and averaged for SS and RS subsets. Results summarized in Table B indicate that the composite internal reliability estimates did not decrease after the removal of temporally inconsistent responders. Interestingly, internal reliability estimates increased slightly after removing temporally inconsistent respondents. Given that this is the first empirical application of the D^2_{ptc} statistic, the current author endorses its usefulness for identifying IER in repeated-measures design. This may be particularly useful for researchers who are limited to observed-score analyses.

Table B - Sample 1 | Summary Composite-Internal and -Temporal Reliability Estimates by Sample and Item-Score Subsets

	Normal score-FTP items		Reverse-score FTP items	
	$(\bar{x})\alpha$	$(\bar{x})$*Test-retest*	$(\bar{x})\alpha$	$(\bar{x})$*Test-retest*
Full ($N = 620$)	.89	.48	.71	.49
Stable ($N = 526$)	.90	.56	.74	.59
