# Supplementary material for: Empirical Tryout of a New Statistic for Detecting Temporally Inconsistent Responders
Source: Front Psychol. 2018 Apr 10;9:518. doi: 10.3389/fpsyg.2018.00518 (PMC5902740; doi:10.3389/fpsyg.2018.00518)
Supplement: Supplementary file 3 [file Data_Sheet_3.PDF]

Online Supplementary Material – APPENDIX C
Application 1 - Latent-Variable Analyses

Because two-wave designs confound measurement error with systematic variance related to substantive change, we supplement the main-test's observed-score analyses with more precise latent variable modeling. That is, we examined the effects of removing D^2_{ptc}-flagged responders based on item response theory (IRT) analyses. Specifically, differential item functioning over time was examined in both full ($N = 620$) and reduced-stable ($N = 526$) samples. Likelihood ration-based statistics for the unidimensional-fitted model are reported in Table C-1 below. Three items (all RS) exhibited evidence of systematic DIF (intercept / location / difficulty parameters) at nominal levels of significance. These findings generally accord with the main text's treatment of temporal reliability of RS items. Notably, after removal of D^2_{ptc}-flagged respondents, one of the RS items no longer exhibited statistical DIF at nominal levels of significance.

Table C1 - Sample 1 (analogous latent analysis) | Summary Uni-DIF Statistics by Slope and Location Parameter Estimates for Time

Item	$X^2_{(location)}$	*Df*	*p*-value	$X^2_{(slope)}$	*Df*	*p*-value
FTP 1	9	6	.17	0.2	1	.68
**FTP 2R**	17.8	6	.01	0.5	1	.47
FTP 3	3.9	6	.68	0	1	.92
FTP 4	1.8	6	.94	0.2	1	.67
FTP 5	4.8	6	.57	0.2	1	.69
FTP 6	6.7	6	.35	0.5	1	.48
FTP 7	7	6	.32	3.2	1	.07
FTP 8	5.3	6	.50	2.6	1	.11
**FTP 9R**	14.3	6	.03	0.4	1	.52
**FTP 10R**	13.8	6	.03	0.6	1	.44

Note. $N = 1{,}240$. Anchored on all items.

In interest to extend evidence for the practical utility of the 'temporal inconsistency' statistic (D^2_{ptc}), three models were compared on global-fit indices. Results displayed in Table B-2 indicate a superior fit of the bifactor model. From the bifactor model, additionally, the latent-stability estimate from FTP's covariance matrix increased, from $\theta\sigma_{2,1} = .70$ to .80. Noteworthy, the RMSEA was maintained at .08, despite removal of approximately 15% of the sample. In contrast, for the two-dimensional model, whereas the latent-stability estimate similarly increased over the unidimensional model, from $\theta_{\sigma 2,1} = .70$ to .84, it incurred model-imprecision as indicated by the elevated *RMSEA* = .11. This is, perhaps, a straightforward demonstration of how temporal stability-estimates may be inflated, but relative to the measurement model error.

Table C-2

Comparative Fit Indices for Longitudinal Models.

Model	[1]M_2 (*df*), *p*-value	-2*ln*L	AIC	BIC	RMSEA
1-Dim	9637.47 (3220), $p < .00$	40917.53	41199.53	41921.85	.04
BiFact	644.49 (127), $p < .00$	40273.23	40439.23	40806.90	.08
2-Dim	1304.21 (137), $p < .00$	41032.69	41178.69	41502.06	.12
Reduced	600.19 (127), $p < .00$	32801.60	32967.60	33321.62	.08

Note. $N = 1{,}240$. -2lnL = -2 log likelihood, AIC = Akiake information criterion, BIC = Bayesian information criterion, 1-Dim= Unidimensional model, Bifact = longitudinal bifactor model, 2-Dim = two-dimensional model, Reduced = D^2_{ptc}-reduced sample with bifactor modeling.
